# Prenatal pembrolizumab exposure as the potential link among recurrent necrotizing enterocolitis, neonatal diabetes mellitus and exocrine pancreatic insufficiency: a case report

**DOI:** 10.1186/s13052-025-02021-8

**Published:** 2025-07-18

**Authors:** Pasquale Castaldo, Luigi Altimari, Maria Grazia Capretti, Giulio Maltoni, Andrea Scozzarella, Vittoria Paoletti, Luigi Corvaglia, Arianna Aceti

**Affiliations:** 1 School of Specialization in Pediatrics, Department of Medical and Surgical Sciences, University of Bologna, Bologna, Italy; 2 Neonatal Intensive Care Unit, IRCCS Azienda Ospedaliero-Universitaria di Bologna, Via Massarenti 11, Bologna, 40138 Italy; 3 Pediatric Unit, IRCCS AOU Bologna, Bologna, Italy

**Keywords:** Necrotizing enterocolitis, Neonatal diabetes mellitus, Pembrolizumab, Case report, Maternal immune checkpoint inhibitor therapy

## Abstract

**Background:**

Necrotizing enterocolitis (NEC) is a serious complication predominantly affecting very low birth weight neonates, characterized by high mortality and long-term morbidity. Neonatal diabetes mellitus (NDM) is a rare condition with diverse etiologies, including genetic predisposition and autoimmune mechanisms. To our knowledge, NEC and NDM have never been previously reported together.

**Case presentation:**

We report the clinical case of a late preterm infant whose mother had received an immune checkpoint inhibitor treatment (pembrolizumab) for metastatic gastric cancer during pregnancy. The infant suffered from severe and recurrent necrotizing enterocolitis (NEC), neonatal diabetes mellitus (NDM) and exocrine pancreatic insufficiency that required a multidisciplinary approach. Prenatal exposure to pembrolizumab might explain the co-occurrence of the three diseases and the complex and unusual medical history of the child, even if the possibility that these events were independent of each other cannot be totally ruled out.

**Conclusion:**

This case suggests the potential association between antenatal pembrolizumab exposure and severe neonatal comorbidities. Furthermore, it highlights the challenge of managing concurrent NEC, NDM and exocrine pancreatic insufficiency in early infancy, emphasizing the need for multidisciplinary care and long-term follow-up to address complex clinical and developmental outcomes.

## Background

Necrotizing enterocolitis (NEC) is one of the most severe complications of premature birth, affecting up to 13% of very low birth weight neonates, with a high rate of morbidity and mortality, especially when surgery is required [1]. Complications include malabsorption and excessive losses from the intestinal stoma leading to failure to thrive, intestinal strictures and adhesions, and short bowel syndrome. Notably, NEC is also associated with long term complications, including an increased risk of neurodevelopmental impairment [2]. 

Neonatal diabetes mellitus (NDM) is an even rarer condition, with a reported incidence of 1 in 90.000-160.000 live births. In multicenter cohort studies, a genetic cause for NDM was identified in up to 80% of cases [3]. Although rare, an autoimmune pathogenesis for NDM should be considered when genetic testing is negative or in the presence of primary or acquired immunodeficiency.

To the best to our knowledge, NEC and NDM have been never described together. We present the clinical case of a late preterm infant with recurrent NEC, NDM, and exocrine pancreatic insufficiency, in whom all the three conditions might be attributed to a single antenatal exposure.

## Case presentation

A small-for-gestational-age female neonate (gestational age 35 + 1 weeks’ and birth weight 1685 g [4], 5-minute Apgar Score 10) was born via scheduled caesarean section due to intrauterine growth restriction and maternal morbidity.

Until the third trimester of pregnancy, her mother had been receiving immune checkpoint inhibitor (ICI) therapy with pembrolizumab to treat a relapse of metastatic poorly differentiated gastric carcinoma, which had been initially diagnosed three years before. Treatment with pembrolizumab was already ongoing at the time of conception: after multidisciplinary team consultations with the family, immunotherapy continuation at the standard dose was decided, and the pregnancy was closely monitored with weekly ultrasound scans. During pregnancy, the mother had also required levothyroxine for gestational hypothyroidism and enoxaparin treatment, that was suspended at 30 weeks gestation for severe idiopathic thrombocytopenia.

At birth, due to the retrieval at prenatal scans of an intestinal dilation of 12 mm, not further characterized, total parenteral nutrition was started; postnatal abdominal X-ray showed only nonspecific and mild dilation of the small bowel, and for this reason, on the third day of life (DOL), enteral nutrition was started and well tolerated.

Since DOL 1, persistent and severe hyperglycemia (maximum blood sugar levels above 400 mg/dl) despite a minimal glucose infusion rate was recorded, early insulin replacement was started and continued at a dosage ranging between 0.05 and 0.1 IU/kg/h. During insulin treatment, the patient showed wide glycemic instability, worsened by acute illness and requiring frequent insulin titration.

On DOL 7, the infant began experiencing frequent vomiting, gastric residual, bloody stools and worsening clinical conditions. Blood tests showed normal values of complete blood count and C-reactive protein, but the abdominal X-ray revealed air-fluid levels in the small bowel, portal venous gas and pneumatosis intestinalis of the distal ileum. Broad-spectrum antibiotic therapy with piperacillin-tazobactam, vancomycin and metronidazole was started, and the patient underwent surgery: twenty centimeters of necrotic distal ileum were resected, a peritoneal catheter was placed, and two ileostomies were made. In the days following surgery, the infant experienced Klebsiella pneumoniae septic shock, characterized by severe thrombocytopenia, leukopenia and metabolic acidosis and requiring inotrope support.

At the end of the second week of life, a second episode of NEC, characterized by intestinal obstruction, peritonitis and fecaloid output from the drainage catheter, occurred; the infant underwent further surgery, which led to the resection of the ileo-cecal valve and of a short, perforated ileal tract.

Early recanalization was not possible, due to poor glycemic control and the occurrence of a second episode of sepsis. Klebsiella pneumoniae was detected on both peripheral venous and central catheter venous samples, with similar time to positivity that excluded catheter-related bloodstream infection. This sepsis episode was managed with initial broad spectrum antibiotic treatment with piperacillin-tazobactam and vancomycin. In consideration of the blood culture results, the sensitivity spectrum and the favorable clinical response, vancomycin was discontinued after few days of treatment.

Ten days after the recurrence of NEC, enteral feeding with a hydrolyzed formula was restarted. Despite achieving an early full enteral feeding, the patient showed failure to thrive and substantial losses of water and electrolytes from the ileostomy, thus requiring high doses of sodium chloride replacement and high daily fluid intakes, to prevent hyponatremia, dehydration and metabolic acidosis. Recanalization was performed at 70 DOL.

Blood tests, which were performed to investigate the cause of persistent hyperglycemia, led to the diagnosis of NDM: low insulin and C-peptide concentrations (C-peptide value of 0.02 ng/ml) were associated with low levels of pancreatic enzymes (i.e. amylase, lipase, fecal elastase) and the antibody panel for type 1 diabetes mellitus was negative (i.e. insulin antibodies, islet cell antibodies, anti-glutamic acid decarboxylase antibodies). No anomaly was detected at the NGS panel including 27 genes for monogenic NDM; abdominal magnetic resonance imaging (MRI) showed mild pancreatic hypoplasia. To improve growth and reduce fecal output, pancreatic enzyme replacement and antisecretory therapy (loperamide) were started. Glycemic control improved after positioning a continuous subcutaneous insulin pump.

During the following months, the infant experienced another episode of bloody stools; no anomalies were detected either in the blood tests or the abdominal X-ray. This episode was managed with a short course of antibiotic therapy and a few days of total parenteral nutrition. Considering the possibility of bile-acids induced colitis, cholestyramine therapy was initiated. The infant was discharged at 165 DOL. Suspecting the occurrence of Small Intestinal Bacterial Overgrowth (SIBO), an antibiotic decontamination therapy with metronidazole was initiated shortly before hospital discharge.

Following the first episode of NEC, multiple areas of intraparenchymal hemorrhage and white-matter damage were detected at cranial ultrasound, which had been normal at birth. Follow-up brain MRI showed severe encephalomalacia. Early neurorehabilitation was started to improve the neurodevelopmental prognosis. Approximately two weeks after discharge, the child experienced epileptic seizures, confirmed by electroencephalography, and an antiepileptic therapy with levetiracetam was started, with effective seizure control.

At present, the patient maintains good glycemic control (last HbA1c < 6.5%, 47 mmol/mol) with ongoing insulin pump treatment (last insulin total daily dose 0.2 u/kg/day); she is still on metronidazole, cholestyramine, and pancreatic enzyme replacement. Weight and linear growth are improving but are still below the third centile for age. No further episodes of enterocolitis have occurred.

## Discussion, limitations and conclusions

To our knowledge, the simultaneous occurrence of severe recurrent NEC, NDM, and exocrine pancreatic insufficiency has not been previously reported. This paper presents an uncommon case suggesting that the three conditions might be linked to a single antenatal exposure.

Severe forms of NEC are mainly experienced by very preterm and very low birth weight infants and are characterized by increased risk of postoperative recurrences, reoperations, perioperative complications, and dependence on parenteral nutrition [5]. NEC can also occur in infants of later gestational age and higher birth weight, although much more rarely and secondarily to predisposing factors such as birth asphyxia, congenital heart disease, anemia, exchange transfusions and drug exposure [6]. 

Pembrolizumab is a humanized IgG4 monoclonal antibody of the ICIs group. It is widely used to treat various adult and pediatric types of cancers, including gastric carcinoma. This molecule acts by inhibiting the programmed death-1 (PD-1) membrane receptor, expressed on the surface of T lymphocytes. PD-1/PD-L1 (programmed death-1 ligand) interaction provides an important “immune checkpoint,” which helps to prevent the immune system from attacking itself. The inhibition of this mechanism, due to pembrolizumab, allows T-cells to increase the intensity of their inflammatory response, improving T-cell mediated killing [7]. Recent studies demonstrates that PD-1/PDL-1 interaction has also a role in fetal-maternal immune tolerance maintenance.PD-1 is expressed by lymphocytes of the maternal decidua throughout gestation, supporting immune balance and preventing autoimmunity. PD-L1, expressed by trophoblasts, decidual macrophages, and stromal cells, reduces T-cell cytokine production, favoring the maternal-fetal tolerance. Theoretically, as an IgG4 class antibody, pembrolizumab can cross the blood placental barrier, especially if administered during the second and third trimesters of pregnancy, determining a potential dysfunction in these pathways. Preclinical data on anti-PD1 antibodies toxicity during pregnancy suggest an increased risk of miscarriage, intrauterine growth restriction, and prematurity [8]. 

**Fig. 1:**
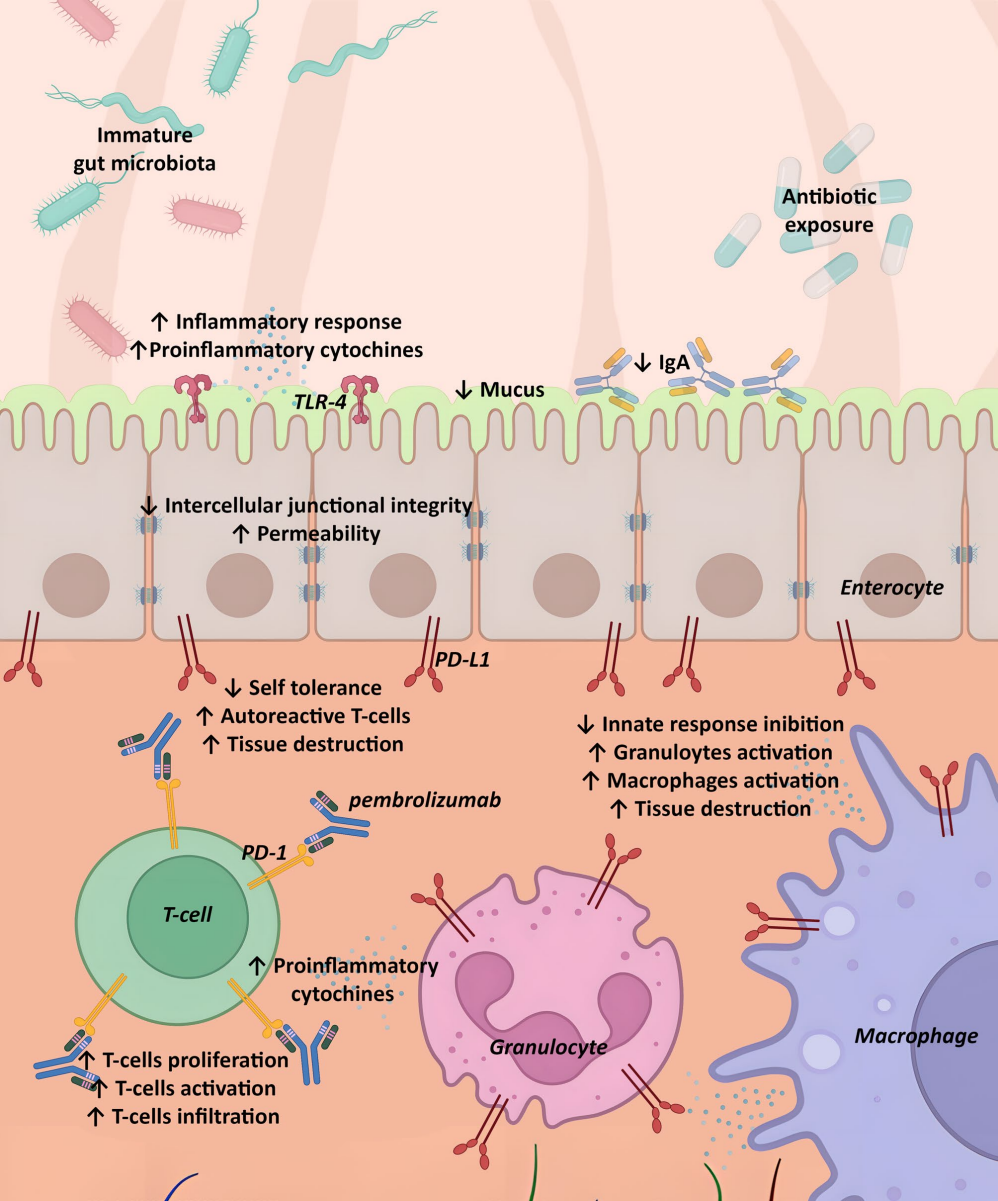
Hypothetical Contribution to the Pathogenesis of Necrotizing Enterocolitis (NEC) in a Premature Neonate Exposed to Pembrolizumab antenatally. In addition to well-established mechanisms predisposing premature infants to NEC (i.e. microbial dysbiosis, the immature intestinal barrier, and the underdeveloped immune system), pembrolizumab-mediated blockade of the PD-1/PD-L1 axis might exacerbate inflammation and tissue destruction. By acting on T cells, the drug reduces self-tolerance, promoting the proliferation, activation, and infiltration of autoreactive T cells. Concurrently, T-cell stimulation amplifies the inflammatory cascade, further activating granulocytes and macrophages

The relationship between PD-1/PDL-1 and NEC has already been studied in a preclinical study. Liu Z. et al. investigated the expression and the possible role of PD-L1 in mice with NEC. The authors showed a worse degree of inflammation and intestinal microscopical and macroscopical changes in knockout PD-L1 mice with NEC. Similarly, in the clinical case reported here, pembrolizumab-induced inhibition of the PD-1/PD-L1 interaction may have exacerbated the inflammatory process underlying NEC, resulting in such severe clinical conditions. Furthermore, a recently reported case described severe immune-related enteritis in a 4-month-old infant exposed to pembrolizumab during pregnancy, presenting with intractable diarrhea and failure to thrive; pembrolizumab dosage on dried blood spot and the assessment of PD-1 expression on intestinal biopsies aided the diagnosis [9]. (Fig. 1)

Alongside the severe NEC condition, the neonate exhibited both endocrine and exocrine pancreatic insufficiency since the first days of life. It is well known that endocrine pancreatic insufficiency is a possible complications of ICIs therapies, occurring in approximately 1% of treated adults and manifesting similarly to type 1 diabetes mellitus (T1DM). The immune response in T1DM, in fact, seems to be like the anticancer immunity generated by immunotherapeutic suppression of PD-1 or its ligand, PD-L1, which normally controls autoimmune reactions. From a clinical perspective, this form of diabetes has a more rapid onset (fulminant in 40–70%) than classical autoimmune T1DM. It can occur as early as 5 days following ICI administration and up to several months after ICI discontinuation [10]. In our case, the infant was exposed to pembrolizumab throughout the entire pregnancy, as the treatment had been discontinued only 21 days before delivery. We could hypothesize a potential role of pembrolizumab in the development of an autoimmune process, occurring gradually in utero or rapidly during the first days of life, from which NDM and exocrine pancreatic insufficiency derived. Despite the negativity of the antibody panel for type 1 diabetes mellitus, the exposition to this drug may have caused an alteration in the trophism of pancreas, which finally resulted in endocrine and exocrine pancreatic insufficiency due to pancreatic hypoplasia. Our hypothesis is supported by a case report of pancreatic exocrine insufficiency involving a 75-year-old man with metastatic melanoma receiving pembrolizumab: blood exams showed low fecal elastase and computed tomography scan displayed pancreas atrophy [11]. 

In the weeks following the first NEC episode, the infant experienced recurrent episodes of enterocolitis, classified as relapses of NEC itself, but not easily interpreted. Differential diagnosis of neonatal enteritis is complex and in the present case included neonatal IBD, bile-induced colitis and SIBO. The association between neonatal IBD and NDM is typical of IPEX syndrome, a primary immunodeficiency disease. This condition, which was excluded through genetic assessment, is linked to a mutation in the FOXP3 gene, located on the X chromosome, which results in T-reg dysregulation, poliendocrinopathy (typically T1DM and autoimmune thyroid disease) and severe enteropathy [12]. 

As for bile induced colitis, it is well documented that reduced ileal length is associated with malabsorption of bile acids, possibly leading to an IBD-like colitis [13]. The excessive presence of bile acids leads to abnormal water and sodium transport, gastrointestinal mucosal damage, increased intestinal motility, diarrhea, and dysbiosis. Treatment with cholestyramine has proven to be effective in managing these symptoms [14]. 

Finally, evidence suggests that the loss or impaired function of the ileo-cecal valve may represent a risk factor for SIBO, a condition characterized by diarrhea, food intolerance, malabsorption and poor growth. The underlying mechanism for this condition could be the migration of colonic microbiota into the ileal portion of the small intestine [15]. 

We are aware that data presented in the present paper are not sufficient to establish a causative role of prenatal exposure to pembrolizumab in triggering NEC and/or pancreatic insufficiency. In addition, it is not possible to exclude that pembrolizumab could be responsible for only part of the clinical picture and that the events described were partially independent of each other. To note, we cannot exclude a genetic etiology for NDM, even if the 27 genes included in the panel account for most known genetic etiologies of neonatal diabetes.

Rather than establishing a causal relationship with absolute certainty, our aim was to describe an unusual clinical case and document the coexistence of three conditions while exploring potential links between them.

NEC still represents one of the most challenging diseases linked with prematurity. In case of recurrent or severe NEC, without any identified risk factor, unusual causes of enterocolitis should be considered, including drug exposure, neonatal IBD, IBD-like colitis and primary immunodeficiency. A multidisciplinary approach is required and is essential to reduce morbidity and mortality and improve long term outcomes.

## Data Availability

Data sharing is not applicable to this article as no datasets were generated or analyzed during the current study.

## Abbreviations

NEC: Necrotizing enterocolitis
NDM: Neonatal diabetes mellitus
DOL: Day of life
ICI: Immune-checkpoint inhibitor
MRI: Magnetic resonance imaging
SIBO: Small intestinal bacterial overgrowth
PD-1: Programmed death-1
PDL-1: Programmed death-1 ligand
T1DM: Type 1 diabetes mellitus
